# E-cigarettes on Instagram: Exploring vape content via an Australian vaping influencer

**DOI:** 10.18332/tid/175619

**Published:** 2024-01-19

**Authors:** Jonine Jancey, Renee N. Carey, Becky Freeman, Tama Leaver, Katharina Wolf, Marilyn Bromberg, Kevin Chai, Stella Bialous, Phoebe Adams, Meghan Mcleod, Kahlia McCausland

**Affiliations:** 1 Collaboration for Evidence, Research and Impact in Public Health, Faculty of Health Sciences, Curtin University, Bentley, Australia; 2 School of Population Health, Curtin University, Bentley, Australia; 3 School of Public Health, University of Sydney, Sydney, Australia; 4 Internet Studies, School of Media, Creative Arts and Social Inquiry, Curtin University, Bentley, Australia; 5 School of Management and Marketing, Curtin University, Bentley, Australia; 6 Law School, The University of Western Australia, Crawley, Australia; 7 School of Nursing, University of California, San Francisco, United States

**Keywords:** e-cigarettes, vaping, social media, Instagram, public health

## Abstract

**INTRODUCTION:**

Mounting evidence suggests that electronic cigarettes (e-cigarettes) are extensively promoted and marketed using social media, including through user-generated content and social media influencers. This study explores how e-cigarettes are being promoted on Instagram, using a case-study approach, and the extent to which Meta’s Restricted Goods and Services Policy (Meta’s policy) is being applied and enforced.

**METHODS:**

We identified the accounts followed by an Australian Instagram influencer who primarily posts e-cigarette-related content. The main foci of these 855 accounts were coded and 369 vaping-focused accounts were identified. These vaping-focused accounts were then further coded by two trained coders.

**RESULTS:**

All (n=369; 100.0%) of the vape content posted by these accounts was positive in sentiment. One-third of the vape accounts (n=127; 34.4%) had a shared focus, indicating that vape content may permeate into other online communities through shared interests. A total of 64 accounts (17.3%) potentially violated Meta’s policy by attempting to purchase, sell, raffle or gift e-cigarette products.

**CONCLUSIONS:**

The findings of this study suggest that pro-vaping information is available and accessible on Instagram. Much of the content identified in this study promoted the purchase or gifting of e-cigarette products and potentially violates Meta’s policy. Greater regulation and/or stronger enforcement of e-cigarette content on social media platforms such as Instagram is necessary to prevent the ongoing promotion of these harmful products.

## INTRODUCTION

Electronic cigarette (e-cigarette) use, particularly among young people, is an ongoing public health concern^1^. The e-cigarette market has proliferated over the past decade, mainly due to use among younger generations^2^. Currently, the global e-cigarette market is estimated at US$24.6 billion, and it is expected to grow at a rate of 3.4% over the next five years^2^. There is mounting evidence to suggest that e-cigarettes are extensively promoted and marketed using social media^3^, with many of the marketing strategies employed designed to attract new consumers. These include the promotion of attractive product flavors and colorful packaging, the use of social media influencers (prominent users with a large number of followers)^4^, and promotion through sociability and lifestyle messages^5^.

The vast majority of e-cigarette-related content on social media platforms such as Instagram, TikTok and Twitter (now known as ‘X’) is pro-vaping^6-8^, with users much more likely to be exposed to positive e-cigarette content than tobacco-control content^7,9,10^. Growing research suggests that the presence of user-generated content (content created and posted by individuals with no formal endorsement)^11^ and marketing related to vaping products on social media can increase the likelihood of young people experimenting with them^5,12,13^. Promotional content and visual posts about e-cigarettes on social media have been linked to increased positive attitudes toward their use^13^. Despite social media content policies restricting the promotion of e-cigarettes^14^, evidence shows that e-cigarette companies and retailers are still using social media to market their products^15^.

The creation, sharing, and interaction with content (including images, text, and links to other social media and websites) is enabled through social media platforms^11,16^. The inherent features of these platforms can be used to great effect in marketing, and offer powerful advantages over traditional broadcast media^16^. For example, companies are able to identify and target users with particular demographic characteristics, connect with these users reciprocally, and gain an understanding of who is most likely to view and interact with their content^11^. Social media also makes possible the gathering of contemporaneous data around how successful their marketing strategies are. Such marketing can include the more traditional paid advertising, enlisting prominent social media users (‘influencers’), and creating free or low-cost brand pages which can be used to distribute marketing content such as links and videos^16^.

Instagram is a visual social media platform owned and operated since 2012 by Meta, the parent company of Facebook^16,17^. It is particularly popular among young adults^18^. According to recent statistics, Instagram has over 2 billion monthly users^19^, with around 31% of those aged 18–24 years and a further 30% aged 25–34 years^18^. Research shows that 82% of Australians using Instagram are willing to use the platform to discover new brands or products, and 89% have taken some form of action after seeing a product or service advertised on Instagram, including following the brand or buying a product online^20^. Further, marketers worldwide reported Instagram as their second most used platform, with 80% of respondents to a global survey stating that they use it to promote their business^21^. This is likely due to the image-focused nature of Instagram making it a particularly effective marketing tool^6^, enabling brands to visually position their products alongside the values, ambitions, and lifestyle of their target audience^15^.

In addition to inorganic or brand-generated content, which is often paid or sponsored, product promotion may also occur through organic or user-generated content, which is not paid for or endorsed by brands^10^. Past research has shown that user-generated content is generally seen by individual followers to be more credible and persuasive than paid or branded content^4^. The use of influencers –prominent social media users who have a large online following and are often paid, or reimbursed in some way, to promote particular brands or products through their social media profiles^4,11^ – may blur this distinction, as influencers also post organic, unpaid content to their profiles^10^. Further, in many cases, branded content is integrated into posts showing everyday activities, obscuring its commercial intent^22^. Many influencers do not disclose their relationships with brands^10^, ignoring advertising guidelines and regulations^23^, and indeed research has shown that such disclosures are negatively related to influencers’ perceived trustworthiness and consumer engagement^22^.

Adding to the complexity, many influencers collaborate with multiple brands and industries, resulting in highly dense and intertwined networks^13,17^. In addition to promoting vaping products, the same influencers may also promote fashion, beauty and lifestyle products. This extends the reach of e-cigarette promotion on social media, as non-e-cigarette-focused audiences can be exposed to e-cigarette content through the influencers they follow. As influencers may have many thousands, or in some cases millions, of followers, the potential reach of this content is vast.

In addition to followers, the number of individuals an influencer follows (their ‘followee count’) is an important determinant of their online influence^24^. Following a higher number of other users may enhance engagement^4^. Following others’ accounts makes them aware of one’s existence and they may follow back (known as reciprocal following), thereby increasing social media reach, potential influence, and influencer status. Thus, following others may be another important way in which content, including e-cigarette posts, are promoted through social media networks. However, research also shows that a high followee count may decrease one’s perceived influence, as these accounts may be viewed as less autonomous and more likely to be susceptible to the views of others^24^. This suggests that having a higher follower-to-followee ratio may have a positive effect on perceived status and influence^25^.

Due to the increasing number of young people using social media, including Instagram, it is clear that stronger regulation is needed to curtail the prevailing promotion of e-cigarette products on these platforms. In Australia, where e-cigarettes are heavily regulated in the ‘real world’^26^, a draft Bill to increase the regulation of e-cigarette promotion and advertising on social media is currently under review^27^. Although Instagram has policies to restrict content promoting e-cigarettes and other novel tobacco products, they are not legally binding. Instagram or its algorithms can decide whether to abide by its own policy^28^, indicating the need for these policies to be stricter. Meta’s Restricted Goods and Services Policy states that it does not support content that promotes the sale of tobacco or e-cigarettes with branded content or advertising, which includes influencers^29^; however, as many influencers do not disclose their brand relationships^10^, the effectiveness of this policy, and Instagram’s enforcement procedures, are unclear.

This study explored how e-cigarettes are being promoted on Instagram using a case study approach. We identified Instagram accounts followed by an Australian-based influencer whose posts are primarily focused on e-cigarette content. This novel case-study approach was adopted, as the automated intelligence data collection method previously used by the researchers^7^ was not accessible for Instagram, as Meta has deliberately changed their application programming interface (API) to prevent researcher access. We assessed the focus of the followed accounts, how e-cigarettes are being promoted on these accounts, and the application of Meta’s Restricted Goods and Services Policy for e-cigarettes.

## METHODS

### Data collection

In October 2022, search terms ‘vaping’ and ‘e-cigarette’ were used to identify an Australian Instagram influencer who primarily posts e-cigarette-related content. In order to identify other e-cigarette-related accounts, we reviewed the accounts this influencer followed. Following others’ accounts make them aware of one’s existence, and they may follow back, increasing social media reach and influencer status. The followed accounts were manually inputted (account name and associated metadata) into an Excel spreadsheet and assigned a number in ascending order. Of the accounts followed, 855 were accessible for analysis (i.e. active and public accounts).

Recorded metadata included the account type (public or private), country of origin, number of accounts followed, number of followers, and number of posts and reels. Country of origin was determined by the mention of a country, town, or flag on the account’s profile page. Up to 40 of the most recent posts from each account were reviewed (some accounts had less than 40 posts at the time of data collection, in which case all posts were reviewed). We also identified whether these accounts were following the influencer back (reciprocal following) by manually reviewing the influencer’s followers.

### Coding frame

An inductive approach informed by extant studies^5,7-9^ was used to develop a coding framework to capture characteristics of account holders (gender and language) and the topic focus of their account (comprising festival, alcohol, food, tattooing, artist, fitness/sport, lifestyle activities, lifestyle interests, and/or vaping content). Up to 40 of the most recent posts (on the day of data collection) for each account were viewed at least five times by two researchers (PA and MM) to identify the focus of the account. A focus was assigned when more than 50% of the posts were centered on the topic.

If the account was identified as vaping-focused (presence of at least 50% vaping and e-cigaretterelated posts), the coding framework then captured additional data. These data included the type of account (individual, retailer, manufacturer, enthusiast, influencer), whether an age restriction to view the account was provided, and links to any external website/s and/or social media accounts, as well as the influencer category. Influencers were defined according to the number of followers they had, whereby non-influencers had <1000 followers; micro-influencers 1000–99999 followers; macro-influencers 100000–999999 followers; and celebrities ≥1000000 followers^30^.

Accounts were also coded for sentiment toward e-cigarette use (positive/negative/neutral), e-cigarette product visibility, the use of e-cigarette brands or logos, e-cigarette product reviews, e-cigarette price promotions, e-cigarette promotion for purchase, e-cigarette customization and tricks, and association with lifestyle and/or business references. The disclosure of e-cigarette branded content or sponsorship was also coded. Each account was also assessed for possible violation of Meta’s Restricted Goods and Services Policy (October 2022)^29^.

The coding framework was tested on 15% of the accounts by two independent coders. This procedure enabled the revision of the coding framework to refine predefined codes, merge codes, and create new codes where required. The coding framework is available as an online Supplementary file Table S1, and the dataset is available on reasonable request.

### Analysis

The modified coding framework was entered into IBM SPSS Statistics (v27). Two researchers (PA and MM) applied the framework to the data, reviewing posts (image and caption) as many times as necessary. Inter-rater reliability was established using Krippendorff alpha, and an average score of α=0.95 was obtained (range: 0.75–1.0), indicating acceptable to perfect agreement^31^. Any disagreements were resolved through discussion between the two coders.

**Figure 1:**
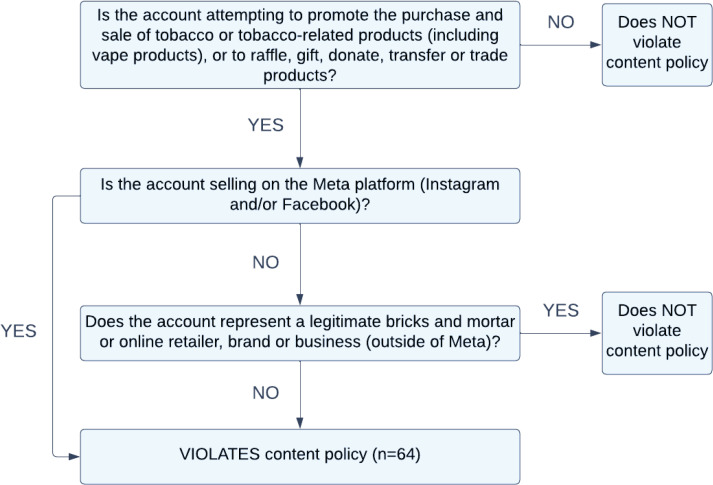
Content policy violations of sampled e-cigarette related Instagram accounts, case study, 2023 (N=64)

The trained coders also reviewed all posts for potential content policy violations using a three-stage process outlined in the coding framework (Supplementary file Table S1) and Figure 1. Content identified as potentially violating Meta’s Restricted Goods and Services Policy^29^ was reviewed by a law academic (MB) for confirmation.

Descriptive statistics were used to summarize account characteristics and metrics (follower and followee counts). The follower-to-followee ratio for each account was calculated by dividing the number of followers by the number of accounts followed, with a ratio of 2:1 used to indicate higher perceived influence^13^. Chi-squared tests were used to show differences in account characteristics, and independent samples t-tests were used to show differences in follower-to-followee ratio by account focus. All tests were two-tailed, and the level of statistical significance was set at p<0.05.

### Ethical considerations

There is consideration by researchers as to whether social media data is public or private. Instagram accounts can be set to public or private by the account holder, and if set to private, requesting followers need to be approved by the account holder. This research only considered publicly available data, which were considered to be secondary data. Even with public accounts, we did not code ephemeral or temporary content (e.g. stories) since their disappearing nature suggests some level of privacy, or at least nonpermanence, is presumed by creators^17^. The study protocol was approved by the Curtin University Human Research Ethics Committee (HR2021-0634), who were informed by Australia’s National Health and Medical Research Council Guidelines Chapter 2.3: Qualifying and waiving conditions for consent^32^.

## RESULTS

In total, 855 accounts were accessible for analysis. These accounts were from a variety of countries, including Australia/New Zealand, United States/Canada, and the United Kingdom (Table 1). The country of origin of 498 accounts (58.2%) could not be determined. The majority of accounts were English-speaking. Just under half of the accounts were held by a group, company, or brand; where the account holder was a person, the majority presented as male (n=373; 80.9%).

**Table 1:** Observed characteristics of all sampled Instagram accounts, case study, 2023 (N=855)

*Characteristics*		*n*	*%*
**Country of origin**			
Australia/New Zealand		131	15.3
United States/Canada		100	11.7
United Kingdom		53	6.2
Other		73	8.6
Could not be determined		498	58.2
**Language**			
English		833	97.8
Other		22	2.2
**Account holder**			
Group, company or brand		394	46.1
Individual		461	53.9
	***Range***	***Mean***	***SD***
Number of followers	16–13300000	165297.4	937403.5
Number of followees	0–7757	1155.2	1484.2
Follower-to-followee ratio	0.1–221000	945.0	8760.3
Number of posts	0–14911	1004.3	1673.8
Number of reels	0–73	4.9	8.3

On average, accounts had more followers (mean=165297.4; standard deviation, SD=937403.5) than followees (mean=1155.2; SD=1484.2) (Table 1). More than half of the accounts (n=487, 57.5%) had a follower-to-followee ratio of 2:1 or greater. Based on follower count, 35.6% (n=304) of the accounts were classified as non-influencers, 51.0% (n=436) micro-influencers, 10.8% (n=92) macro-influencers, and 2.7% (n=23) celebrities.

The majority of accounts (n=465; 54.4%) had a single focus, with the main focus being everyday lifestyle activities including leisure and social activities (n=248; 29.0%), lifestyle interests including entertainment and hobbies (n=179; 20.9%), and artists such as musicians or actors (n=146; 17.1%).

Almost half of all accounts (n=369; 43.2%) were identified as being focused on vaping content. One-third of these vape-focused accounts (n=123; 33.3%) reciprocally followed the originally identified influencer, compared with three-quarters of the non-vape-focused accounts (n=371; 76.3%) [χ^2^(1)=159.0, p<0.001].

One-third (n=127; 34.4%) of these vape-focused accounts also had at least one other focus, and 57 (15.4%) had two or more additional foci. Vape accounts held by micro-influencers (n=162; 72.3%) and macro-influencers (n=22; 81.5%) were more likely to have a sole rather than shared focus [χ^2^(2)=21.6, p<0.001].

Of those vape accounts with a shared focus, the most common additional foci were lifestyle activities, including travel and other social activities with friends and family (n=67; 18.2%), and lifestyle interests, including hobbies such as photography and sports (n=59; 16.0%). Other less common shared foci included food (n=32; 8.7%), festivals (n=26; 7.0%), alcohol (n=22; 6.0%), tattooing (n=14; 3.8%), fitness and sport (n=8; 2.2%), and artists (n=7; 1.9%).

Accounts posting vape content (mean=145.9; SD=147.8) had a significantly lower follower-to-followee ratio than accounts that did not post any vape content (mean=1556.0; SD=528.4) [t(845)=2.3, p=0.020]. A total of 209 vape-focused accounts (59.7%) had a ratio of 2:1 or greater, signifying higher perceived influence.

Of those accounts posting vape content, approximately one-third (n=133; 36.0%) were identified as manufacturers of e-cigarette products, 79 (21.4%) as vape enthusiasts or advocates, and 76 (20.6%) as retailers or vendors of e-cigarette products. Over two-thirds of accounts (n=251; 68.0%) posting vape content were classified as influencer accounts, with 224 (60.7%) being micro-influencers and 27 (7.3%) macro-influencers. All (n=369; 100.0%) vape content posted on these accounts was positive in sentiment.

Over half of the vape-focused accounts (n=222; 60.2%) included a vape-related term in either their username or bio. The majority of vape accounts (n=186; 50.4%) included a link to another page or website containing vape-related content, and 135 (36.6%) linked to online vape shops or businesses. In addition, 161 (43.6%) accounts referenced a business website or page relating to vape products, for example, encouraging users to ‘follow this page for more’. Age restrictions or warnings were present on only a small number of account pages (n=18; 4.9%) or linked web pages (n=55; 14.9%).

Table 2 shows the number of accounts featuring observed characteristics along with the associated number of followers and followees. E-cigarette products were visible on the majority of accounts (n=328; 88.9%), including e-liquids (n=238; 64.5%) and other vape products such as coils, filters, and vaping accessories (n=266; 72.1%). E-cigarette brands or logos were visible on over three-quarters of accounts (n=294; 79.7%); however, no accounts disclosed paid partnerships or sponsorships. Those accounts showing e-cigarette products and brands had a higher mean number of followers (mean=29303.3; SD=74105.6) than those not showing products and brands (mean=12387.9; SD=27236.2), indicating greater reach, although this difference was not statistically significant [t(367)=1.4, p=0.148].

**Table 2:** Observed characteristics of sampled e-cigarette related accounts on Instagram, case study, 2023 (N=369)

*Characteristics*	*n*	*%*	*Followers*	*%*	*Following*	*%*
	369		10119391		484372	
**Products**						
E-cigarette products visible	328	88.9	9611487	95.0	446571	92.2
E-liquids visible	238	64.5	6360038	62.8	326943	67.5
Other vape products visible	266	72.1	7016300	69.3	345714	71.4
Brand or logo visible	294	79.7	8340203	82.4	380735	78.6
**Content**						
Product review	117	31.7	2682485	26.5	170918	35.3
Promotes purchase of vape product	231	62.6	7918431	78.3	294144	60.7
Includes a price promotion for vape product	83	22.5	3290974	32.5	112805	23.3
Customization of vape products and/or juices	182	49.3	5163866	51.0	217401	44.9
Vape tricks	53	14.4	2002247	19.8	82136	17.0
Associates vape products with lifestyle	72	19.5	1868056	18.5	95040	19.6
Includes disclosure of sponsored content	0	0.0	0	0.0	0	0.0
References a business associated with vape products	161	43.6	3817546	37.7	229183	47.3

E-cigarette product reviews (n=117; 31.7%) and the promotion of vape products for purchase (n=231; 62.6%) were common on the identified accounts, with just under one-quarter of accounts (n=83; 22.5%) also including a price promotion, discount, offer or giveaway for vaping products (Table 2). Those accounts that promoted e-cigarettes for purchase had a significantly higher mean number of followers (mean=34278.9; SD=83141.6) than those not promoting e-cigarette purchases (mean=15949.0; SD=39795.1) [t(367)=2.4, p=0.016].

The content of accounts included references to customizing or modifying vape products (n=182; 49.3%), vape tricks (n=53; 14.4%), and a general positive lifestyle associated with vape products (n=72; 19.5%).

A total of 64 accounts (17.3%) were found to violate Meta’s Restricted Goods and Services policy. Accounts that were found to be in potential violation of the content policy had a lower proportion of followers (n=753341; 7.4%) and a lower follower-to-followee ratio (mean=59.9; SD=214.7) than those that did not appear to violate the policy (mean=163.7; SD=1001.7); however, this was not statistically significant [t(365)= -0.8, p=0.414].

Reasons for policy violation are outlined in Figure 1. All violations related to attempting to promote the purchase or sale of tobacco or tobacco-related products or to raffle, gift, donate, transfer, or trade products. While a large number of the accounts promoting the purchase or gifting of tobacco products were legitimate brick-and-mortar or online entities (n=254; 89.1%), a number of them (n=33; 13.0%) were also attempting to sell or gift products using the Meta platform, which is a violation of the policy. An additional 31 accounts were promoting the purchase or gifting of tobacco products and were not legitimate brick-and-mortar or online entities, violating content policy. Of those, 22 (71.0%) were using the Meta platform to sell or gift products.

## DISCUSSION

This study identified 369 vaping-focused Instagram accounts followed by an Australian influencer, in order to explore how e-cigarettes are being promoted on Instagram, and how Meta’s Restricted Goods and Services Policy is being applied. All of these followed vape-focused accounts (n=369) expressed a positive sentiment towards e-cigarettes, consistent with previous research investigating the portrayal of e-cigarettes on various social media platforms including Instagram^6-8^. In addition, e-cigarette products including e-liquids and other products such as coils and filters, were visible on the majority of these accounts, and in many cases, the brand or logo of the product was also shown.

Over half of the vape-focused accounts identified in the current study included a vape-related term such as ‘vape’, ‘e-cigarette’ or ‘juice’ in either their username or bio, indicating the accessibility of vape-related content on Instagram. The majority of vape-focused accounts included a link to another page or website with vape-related content or an online vape shop or business. While this is not a violation of Meta’s Restricted Goods and Services Policy provided the business is a legitimate brick-and-mortar or online entity^29^, the proliferation of these links shows how the purchase of vape products may be facilitated through social media platforms. This is especially concerning as recent research in the US^33^ and Australia^34^ shows that around one-third of adolescents who purchase vaping devices report using online sources including social media to do so. Further, product reviews and price promotions were relatively common on the vape accounts identified. This type of content has previously been shown to be common on social media^3^, and may act to influence potential customers to purchase vape products and encourage brand loyalty^35^.

In total, we found that 64 accounts potentially violated Meta’s Restricted Goods and Services Policy^29^, consistent with past research showing that the violation of social media platform policies is common^10,14^. In all cases, the violations were related to the attempt to promote the purchase, sale, gifting, donation, or trade of e-cigarette products. While promoting the purchase or gifting of e-cigarette products through a legitimate brick-and-mortar or online business is not in itself a violation of policy, promoting purchases through the Meta platform is a violation. Adding to the complexity, there is no definition of a legitimate business in the policy wording, making it difficult to determine where a violation has occurred. A majority of the violating accounts were found to be using the Meta platform to sell or gift e-cigarette products. In addition, 31 accounts that were promoting the purchase or gifting of these products were not legitimate businesses. This highlights the shortcomings of allowing Meta and other social media companies to determine their own policies and the observance of these^28^. These data point to a clear need for government regulation of e-cigarette advertising, promotion, and sponsorship as outlined in the draft Public Health Tobacco and Other Products Bill 2023 currently under review in the Australian parliament, which now separates advertising of traditional tobacco and e-cigarettes and also makes it clear that advertising e-cigarettes online is not permitted^27^. The Bill states an e-cigarette advertisement is classified as any form of communication, recommendation or action that promotes or is likely to promote (whether directly or indirectly) vaping, an e-cigarette product or the use of such a product.

We also found a lack of sponsorship disclosures, so it is unclear how many accounts had paid partnerships with e-cigarette brands, although a majority of accounts showed e-cigarette product brands or logos. This is consistent with past research showing minimal compliance with sponsorship disclosure regulations among vaping influencers on Instagram^10^. This is likely a result of Meta’s policy prohibiting e-cigarettes from being promoted with branded content and once again highlights the shortcomings of social media content guidelines and policies. While influencer industry guidelines and social media platform policies require transparency and disclosure, these findings and past research show that such promotions are frequently not labelled or disclosed^7,10^. Such disclosure is also likely hampered by the unclear definition of promotion in social media content policies, with the lines between the presence of vaping content (which is generally allowed), the promotion of vaping products (which is allowed in some forms), and paid advertising (which is not allowed) being blurred. This means that many accounts and posts exist in the grey area of this spectrum. There is a clear need for content policy wording to capture the promotion of e-cigarettes more accurately, particularly in relation to influencer marketing, and for more emphasis to be placed on the enforcement of these policies^7^.

A substantial number of the vape-focused accounts in the current study had at least one other focus, most commonly lifestyle activities and lifestyle interests. This is consistent with research showing that in addition to promoting vaping products, influencers may also promote other products such as fashion and lifestyle^13^. E-cigarette-related content may permeate into other online communities through these shared interests, increasing its reach. In addition, we found that those accounts that were reciprocally following the originally identified e-cigarette influencer were more likely to be non-vape-focused. This reciprocal following may be another way that e-cigarette-related content can spread through other online communities.

### Limitations

The generalizability of our findings may be limited by our sampling method. As Instagram did not allow the automated intelligence data collection method that we have previously used to collect data from other social media platforms^7^, we adopted a novel case-study approach whereby we were able to identify the followees of one Australian influencer who primarily posts e-cigarette-related content. As a result, it is not known how well these sampled accounts may represent the broader population of Instagram account holders or vape-focused accounts, but they do provide valuable insights into this Instagram content. In addition, by only reviewing the most recent 40 posts of each account, we may have incorrectly assigned the focus of these accounts or missed other important content. Not coding ephemeral media such as Stories (short-lived posts that automatically disappear after 24 hours) may also have impacted the coding of accounts. However, this was a practical decision. Our analysis was descriptive in nature only and did not take into account any potential confounding variables. Further, we are unable to draw any conclusions about the effects of the posts and accounts studied here, although previous research has suggested that e-cigarette-related social media content leads to the normalization of e-cigarette use, particularly among young people^12^.

## CONCLUSIONS

Our study demonstrates the pervasiveness of positive e-cigarette promotion on Instagram, the challenges around interpretation of Meta’s Restricted Goods and Services Policy, enforcement of that policy, and the mechanisms that enable access to and purchasing of e-cigarette products. Much of the identified content promoted the purchase or gifting of e-cigarette products, which openly violates Meta’s Policy and highlights the shortcomings of allowing Meta and other social media companies to determine their own policies and manage the associated content. In addition, this content is likely permeating into other online communities through shared foci and reciprocal following.

Greater regulation of e-cigarette content on social media platforms such as Instagram is necessary to prevent the ongoing promotion of these harmful products. Regulations, such as the draft Public Health (Tobacco and Other Products) Bill 2023 currently under review in the Australian parliament, will hopefully make it clear that the promotion of e-cigarettes online is not permitted; however, the exact regulations that may be passed are still unknown^27^. Regulations that are enacted must be fit-for-purpose, promoted, and enforced to enable the prevention of the ongoing positive promotion of these products.

Moving forward, there needs to be transparent and independent assessments of the impact of social media products and services on social media users, assessment of the content policy and its enforcement, assessment of the impact of the proposed regulations, if passed by the Australian Parliament, prioritizing of the health and safety of social media users by these digital companies, and exploration of ways to more effectively manage harmful products such as e-cigarettes in the social media environment.

## Data Availability

The data supporting this research are available from the authors on reasonable request.
