# Supplementary material for: E-cigarettes on Instagram: Exploring vape content via an Australian vaping influencer
Source: Tob Induc Dis. 2024 Jan 19;22:10.18332/tid/175619. doi: 10.18332/tid/175619 (PMC10798224; doi:10.18332/tid/175619)
Supplement: Supplementary file 1 [file TID-22-19-s1.pdf]

Supplementary Table S1. Codebook used to categorise sampled Instagram accounts

CODES AND DEFINITIONS	CODING RULES
**Account name**	Insert account name.
**Account type**<br>1 = private account<br>2 = public account<br>3 = account does not exist	Is the account private, public, or does not exist?<br>Private = not accessible<br>Public = accessible<br>Account cannot be found
**Country**<br>1 = United States/Canada<br>2 = South America<br>3 = United Kingdom<br>4 = Australia/New Zealand<br>5 = Europe<br>6 = Asia<br>7 = Other<br>8 = Cannot identify	What is the country of origin?<br>Identified by flag or written in text (as no location field in Instagram profiles)
**Following**	What is the number of accounts being followed?
**Followers**	What is the number of account followers?
**Account following back**<br>1 = yes<br>2 = no	Is the followed account following back?
**Influencer category**<br>1 = not an influencer<br>2 = micro influencer<br>3 = macro influencer<br>4 = celebrity influencer	What level of influencer are they?<br>Not an influencer = <1,000 followers<br>Micro-influencers = >1,000 – 99,999 followers<br>Macro-influencers = >100,000 – 999,999 followers<br>Celebrities = >1,000,000 followers [37]
**Posts**	What is the total number of posts?
**Reels/videos**	What is the total number of reels/videos?
ACCOUNT HOLDER CHARACTERISTICS	
**Male**<br>1 = yes<br>2 = no	Does the account holder identify as male? (identified male/masculine pronouns and inferred visually)
**Female**<br>1 = yes<br>2 = no	Does the account holder identify as female? (identified female/feminine pronouns and inferred visually)
**Non-binary**<br>1 = yes<br>2 = no	Does the account holder identify as non-binary (determined by pronoun in bio)
**Other**<br>1 = yes<br>2 = no	Is the account a group, company or brand that does not identify a person?
**English speaking**<br>1 = yes<br>2 = no	Is the account language English? (based on text)
**Other language**	If another language, record the language.

MAJOR FOCUS OF ACCOUNT	Determined on review of up to 40 of the most recet posts. More than 50% of posts are focussed on the topic.
**Festival**<br>1 = yes<br>2 = no	Are the posts focused on a festival (alcohol, food, music, entertainment, vape expo)?
**Festival type**	Describe in text
**Festival category**<br>1 = alcohol<br>2 = food<br>3 = music<br>4 = entertainment<br>5 = vape expo	What is the focus of the festival?<br>**Alcohol** including beer, wine, spirits.<br>**Food** including BBQ, pub, café/restaurant and homestyle foods.<br>**Music** including DJs, country, pop, electronic and indie genres.<br>**Entertainment** including tattoo expo, wedding expo.<br>**Vape expo** = spaces for mass gatherings of people to review and purchase vape products.
**Number of festival posts**	How many posts are focussed on the topic?
**Alcohol**<br>1 = yes<br>2 = no	Are the posts focused on alcohol?
**Alcohol type**	Describe in text
**Alcohol category**<br>1 = beer<br>2 = wine<br>3 = spirits	What alcohol type is the focus?<br>**Beer** including craft beer, lager, ale, cider, breweries.<br>**Wine** including all wine types and vineyards.<br>**Spirits** including gin, whisky, vodka, tequila, sake, cocktails, and distilleries.
**Number of alcohol posts**	How many posts are focussed on the topic?
**Food**<br>1 = yes<br>2 = no	Are the posts focused on food?
**Food type**	Describe in text
**Food category**<br>1 = sweet<br>2 = savoury	Are the posts focused on sweet food (cakes, desserts, confectionery) or savoury food (meats, fish, vegetables)?
**Food setting**<br>1 = café/restaurant<br>2 = pub food<br>3 = home food	What type of food setting is the focus?<br>**Café/restaurant** including brunch, breakfast, coffee, ice cream, fine dining, various cuisines, and fast foods.<br>**Pub food** including burgers, chips, pies, pizza.<br>**Home food** including chutney, peanut butter, roast, snacks, groceries, home delivery such as Hello Fresh.
**Number of food posts**	How many posts are focussed on the topic?
**Tattooing**<br>1 = yes<br>2 = no	Are the posts focussed on tattoos (e.g., tattooist, tattoo shop, business, tattoo design)?

**Number of tattoo posts**	How many posts are focussed on the topic?
**Artist**<br>1 = yes<br>2 = no	Are the posts focused on an artist?
**Artist type**	Describe in text
**Artist category**<br>1 = musician<br>2 = comedian<br>3 = actor<br>4 = creatives	What type of artist is the focus?<br>**Musician** including DJ, bands, solo singers, cellists, pianists.<br>**Comedian:** entertainer focussed on making people laugh.<br>**Actor:** stage and theatre.<br>**Creatives** including photographers, authors, podcasters, graphic designers, interior designers, pottery, jewellers, patch makers, painters, drawers, and make-up artists.
**Number of artist posts**	How many posts are focussed on the topic?
**Fitness/sport**<br>1 = yes<br>2 = no	Are the posts focused on fitness and sport?
**Fitness/sport type**	Describe in text
**Fitness/sport category**<br>1 = team sport<br>2 = individual	What type of sport is the focus?<br>**Team sport:** soccer, rugby league, Australian Rules, basketball, hockey.<br>**Individual sport:** weightlifting, pole vaulting, gym, running, Formula One driving, horse riding, BMX, motorbike, pilates.
**Number of fitness/sport posts**	How many posts are focussed on the topic?
**Lifestyle activities**<br>1 = yes<br>2 = no	Are the posts focussed on everyday lifestyles: walking dogs, nature, family, friends?
**Lifestyle type**	Describe in text
**Lifestyle activity category**<br>1 = friends/family<br>2 = leisure	What type of lifestyle activity is the focus?<br>**Friends/family:** gatherings, socialising, pets<br>**Leisure:** holidays, outings, gardening, walking dogs and celebrating holidays (Christmas etcetera).
**Number of lifestyle activity posts**	How many posts are focussed on the topic?
**Lifestyle interests**<br>1 = yes<br>2 = no	Are the posts focused on lifestyle interests (e.g., beauty/skin care products, fashion, bikes, skateboards)?
**Lifestyle interests type**	Describe in text
**Lifestyle interest category**<br>1 = entertainment<br>2 = hobbies	What lifestyle interests are they focussed on?<br>**Entertainment** including listening to music, movies, memes.<br>**Hobbies** including boating, fishing, skateboarding, bikes, cars, amateur photography.

**Number of lifestyle interest posts**	How many posts are focussed on the topic?
**Other**<br>1 = yes<br>2 = no	Are the posts focussed on other topics?
**Other type**	Describe in text
**Other category**<br>1 = entertaining<br>2 = business	What other interests are they focussed on?<br>**Entertaining:** memes and quotes (memes being a photo accompanied by text – usually humorous)<br>**Business:** official business accounts
**Number of other posts**	How many posts are focussed on the topic?
**Vape content**<br>1 = yes<br>2 = no	Are the posts focussed on vaping content (e.g., displays, advertises, promotes vape/e-cigarette product content)?
**Number of vaping posts**	How many posts are focussed on the topic?
TYPE OF ACCOUNT	ONLY FOR VAPING-RELATED ACCOUNTS
**Person/individual**<br>1 = yes<br>2 = no	Is the account run by an everyday person?<br>[small number of followers]
**Retailer/vendor**<br>1 = yes<br>2 = no	Is the account an outlet that sells vape/e-cigarette related products?
**Manufacturer**<br>1 = yes<br>2 = no	Is the account a manufacturer of vape/e-cigarette related products?
**Vape enthusiast/advocate**<br>1= yes<br>2 = no	Content promotes e-cigarettes but is not trying to sell e-cigarettes or related products.<br>*Can be an everyday person.*
VAPE ACCOUNT DETAILS	
**Vape term in username**<br>1 = yes<br>2 = no	Does the account contain a vape related term in the username (e.g., ‘vape’, ‘e-cigarette’, ‘coils’, ‘juice’, ‘mod’, etc.)?<br>[can be separate or not separate word]<br>* at the time these were terms banned on Instagram
**Name if yes**	Record name.
**Vape related term in bio**<br>1 = yes<br>2 = no	Does the account bio contain a vape related term in the username (e.g., ‘vape’, ‘e-cigarette’, ‘coils’, ‘juice’, ‘mod’, etc.)?<br>* at the time these were terms banned on Instagram
**Term if yes**	Record term.
**Age restriction listed in bio**<br>1 = 18 years age restriction<br>2 = 21 years age restriction<br>3 = no age restriction	Is there a stated age restriction on the homepage?

**Bio contact details**<br>1 = yes<br>2 = no	Are there contact details in the bio (e.g., email, phone number, DM, WhatsApp)?
**Link in bio**<br>1 = yes<br>2 = no	Is there a link to Instagram, YouTube, Facebook, Linktree, or website in bio?
**Link to vape products**<br>1 = yes<br>2 = no<br>3 = unsure as link broken	Is there a link/website with vape related content?<br>Link broken may indicate vape account taken down; non-valid license for webpage.
**Record online vape shop link**	Insert link.
**Link to personal blog**<br>1 = yes<br>2 = no<br>3 = unsure as link broken	Is there a link to a personal blog?<br>If the account has been coded as yes [1], then the link that they have provided in the bio will link to vape related content.
**Record personal blog link**	Insert link.
**Link to Instagram account**<br>1 = yes<br>2 = no<br>3 = unsure as link broken	Is there a link to an Instagram account?<br>If the account has been coded as yes [1], then the link that they have provided in the bio will link to vape related content.
**Record Instagram link**	Insert link.
**Link to Facebook account**<br>1 = yes<br>2 = no<br>3 = unsure as link broken	Is there a link to a Facebook account?<br>If the account has been coded as yes [1], then the link that they have provided in the bio will link to vape related content.
**Record Facebook link**	Insert link.
**Link to YouTube account**<br>1 = yes<br>2 = no<br>3 = unsure as link broken	Is there a link to a YouTube account?<br>If the account has been coded as yes [1], then the link that they have provided in the bio will link to vape related content.
**Record YouTube link**	Insert link.
**Age warning on linked web page**<br>1 = yes<br>2 = no<br>3 = N/A	Is there an age warning (18 or 21 years) when accessing the linked page?<br>[N/A = does not link to vape related products]
VISUALS	
**Vape products visible**<br>1 = yes<br>2 = no	Are e-cigarette products visible among the first 40 posts of the homepage?
**E-liquid products visible**<br>1 = yes<br>2 = no	Are there e-liquid products visible among the first 40 posts of the homepage?
**Other vape products**<br>1 = yes<br>2 = no	Are there coils, filters, mods, vaping accessories, and/or heat-not-burn products visible among the first 40 posts on the homepage?

**Product review**<br>1 = yes<br>2 = no	Are there opinion on specific vaping products among the first 40 posts on the homepage?
**Promote vape products for purchase**<br>1 = yes<br>2 = no	Is there promotion of vaping products for purchase among the first 40 posts on the homepage?<br>[where to purchase, product reviewer promotes product and where to purchase]
**Price promotion**<br>1 = yes<br>2 = no	Are there coupons, discounts, offers, multi-buys, giveaways among the first 40 posts on the homepage?
**Customisation**<br>1 = yes<br>2 = no	Is there customising or modifying vape products (e.g., changing coils, changing pods, changing e-juice) among the first 40 posts on the homepage?
**Vape tricks**<br>1 = yes<br>2 = no	Are there vaping tricks using aerosol plumes among the first 40 posts on the homepage?
**Pro-vaping**<br>1 = yes<br>2 = no	Is there pro-vaping messaging (e.g., 'Vaping can help people quit smoking') among the first 40 posts on the homepage?<br>Do the 40 posts reference advocacy for encouraging the use of vapes/e-cigarettes?<br>[overt = e-cigarettes healthier than cigarettes OR covert = open use of product or advertising products]
**Anti-vaping**<br>1 = yes<br>2 = no	Is there discouraging messaging (encouraging cessation, prevention, reference to anti-vaping advocacy activity) among the first 40 posts on the homepage?
**Sentiment**<br>1 = positive<br>2 = negative<br>3 = neutral	What type of sentiment is present among the first 40 posts on the homepage?<br>**Positive:** promoting/encouraging e-cigarette use.<br>**Negative:** discouraging/deterring e-cigarette use.<br>**Neutral:** presents a balanced view
**Brand visible**<br>1 = yes<br>2 = no	Is there product brands or logos visible among the first 40 posts on the homepage?
**Other lifestyle associations**<br>1 = yes<br>2 = no	Are the vape products associated with a lifestyle (e.g., appearing to be rich, beautiful, healthy, happy, positive) visible among the first 40 posts on the homepage?
**Branded content**<br>1 = yes<br>2 = no	Is there an indication that the account is sponsored or partnered with a business or influencer?<br>[e.g., 'paid partnership with…' OR #sponsored #ad]
**Business affiliation**<br>1 = yes<br>2 = no	Do the posts reference following a business page or linking to a business website related to vape/e-cigarette products?<br>[e.g., 'follow this page for more', 'follow the link in bio to purchase']

CONTENT POLICY	Content policy assessment is taken from Meta's Restricted Goods and Services Policy[35]
**1A. Content policy**<br>1 = yes<br>2 = no<br>**If yes, may violate content policy**	Is the account (individual, manufacturer or retailer) attempting to promote the purchase or sale of tobacco or tobacco-related products?
**1B. Content policy**<br>1 = yes<br>2 = no<br>**If yes, may violate content policy**	Is the account (individual, manufacturer or retailer) attempting to raffle, gift, donate, transfer or trade tobacco or tobacco-related products?<br>* can include discount and two-for-one deals
**2A. Content policy**<br>1 = yes<br>2 = no<br>**If yes, does not violate content policy**	Is the account attempting to buy, sell or trade, or donate or gift tobacco or tobacco-related products posted by a page, group, or Instagram profile representing legitimate brick-and-mortar entities, including retail businesses, websites or brands, or a private individual sharing content on behalf of legitimate brick-and-mortar entities (essentially off Instagram)?<br>* determined if they provide a web link or provide official page in bio
**2B. Content policy**<br>1 = yes<br>2 = no<br>**If yes, does violate content policy**	Is the account attempting to buy, sell or trade, or donate or gift tobacco or tobacco-related products posted by a private/individual/manufacturer selling on Instagram platform (e.g., DM link or email address; links to other Instagram and/or Facebook sites)?
**3A. Content policy**<br>1 = yes<br>2 = no<br>**If yes, does not violate content policy**	Does the content refer to tobacco or tobacco-related products that will be exchanged or consumed on location at an event, restaurant, bar, party, and so on?
**3B. Content policy**<br>1 = yes<br>2 = no<br>**If yes, does violate content policy**	Does the account ask for tobacco or tobacco-related products?
**Violates content policy**<br>1 = yes<br>2 = no	Does the account violate content policy?<br>[considering all the content policy assessment]
**Reason for violation**	Summarise by providing reason for content policy violation.

© 2024 Jancey J. et al.
